# Bistable Hofmann-Type Fe^II^ Spin-Crossover Two-Dimensional Polymers of 4-Alkyldisulfanylpyridine for Prospective Grafting of Monolayers on Metallic Surfaces

**DOI:** 10.1021/acs.inorgchem.1c01010

**Published:** 2021-05-28

**Authors:** Rubén Turo-Cortés, Francisco Javier Valverde-Muñoz, Manuel Meneses-Sánchez, M. Carmen Muñoz, Carlos Bartual-Murgui, José Antonio Real

**Affiliations:** † Instituto de Ciencia Molecular/Departamento de Química Inorgánica, Universidad de Valencia, Catedrático Beltrán Martínez 2, 46980 Paterna, Valencia Spain; ‡ Departamento de Física Aplicada, Universitat Politècnica de València, Camino de Vera S/N 46022 Valencia, Spain

## Abstract

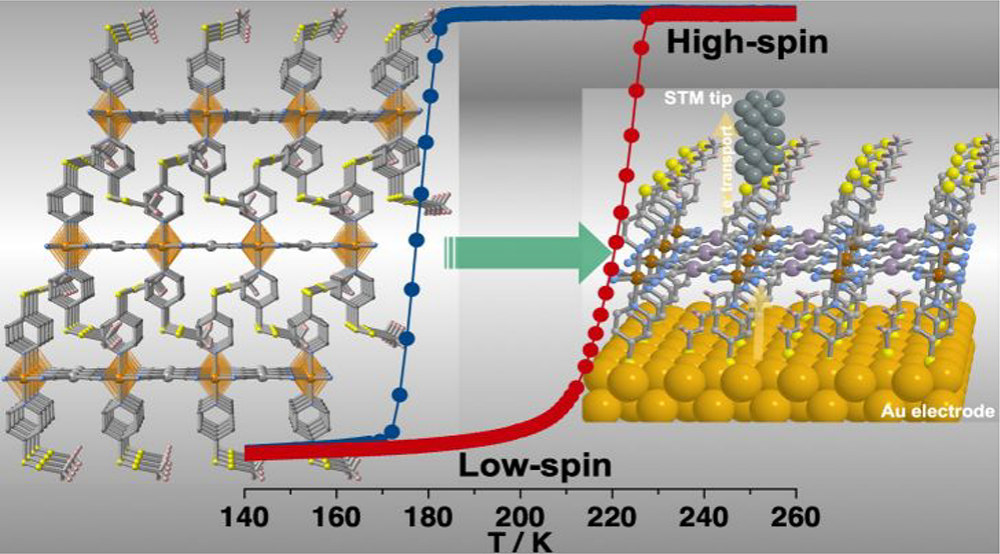

Aiming at investigating the suitability of Hofmann-type two-dimensional (2D) coordination polymers {Fe^II^(L_ax_)_2_[M^II^(CN)_4_]} to be processed as single monolayers and probed as spin crossover (SCO) junctions in spintronic devices, the synthesis and characterization of the M^II^ derivatives (M^II^ = Pd and Pt) with sulfur-rich axial ligands (L_ax_ = 4-methyl- and 4-ethyl-disulfanylpyridine) have been conducted. The thermal dependence of the magnetic and calorimetric properties confirmed the occurrence of strong cooperative SCO behavior in the temperature interval of 100–225 K, featuring hysteresis loops 44 and 32.5 K/21 K wide for Pt^II^-methyl and Pt^II^/Pd^II^-ethyl derivatives, while the Pd^II^-methyl derivative undergoes a much less cooperative multistep SCO. Excluding Pt^II^-methyl, the remaining compounds display light-induced excited spin-state trapping at 10 K with *T*_LIESST_ temperatures in the range of 50–70 K. Single-crystal studies performed in the temperature interval 100–250 K confirmed the layered structure and the occurrence of complete transformation between the high- and low-spin states of the Fe^II^ center for the four compounds. Strong positional disorder seems to be the source of elastic frustration driving the multistep SCO observed for the Pd^II^-methyl derivative. It is expected that the peripheral disulfanyl groups will favor anchoring and growing of the monolayer on gold substrates and optimal electron transport in the device.

## Abstract 2

We report a series of layered iron(II) Hofmann coordination polymers that display thermal- and light-induced spin-crossover (SCO) behaviors featuring wide thermal hysteresis. The axial pyridine-type ligands, functionalized with peripheral sulfur-rich substituents, make these polymers potential candidates for processing and integrating them as SCO monolayers in spintronic transport devices.

## Introduction

Bistable molecular materials with switchable properties are appealing candidates for developing technological applications, e.g., sensors for information storage. Iron(II) spin crossover (SCO) complexes afford excellent examples of molecular bistability, because they reversibly switch between the high-spin (HS, t_2g_^4^e_g_^2^) and low-spin (LS, t_2g_^6^e_g_^0^) electronic states in response to a variety of external stimuli such as temperature, pressure, light, adsorption of analytes or extrinsic phase transitions. This is particularly true when the spin changing centers are strongly coupled to each other, since the spin state change manifests cooperatively conferring hysteretic behavior (memory effect) to the magnetic, optical, structural, mechanical, and electric properties associated with the material.^1^

The SCO research is a very active and multidisciplinary field that spreads in many complementary directions. The synthesis and characterization of interesting mononuclear, polynuclear, and one-dimensional to three-dimensional (1D–3D) polymeric SCO systems has increased exponentially during the last two decades, affording new SCO behaviors^2^ which, in turn, have inspired new sophisticated physical techniques and theoretical models.^1e,3^ To engineer new multifunctional materials where the SCO synchronically interplays with other relevant physicochemical properties—e.g., porosity (host–guest chemistry), liquid crystalline properties, crystal-to-crystal phase transitions, luminescence or chirality—in a synergetic fashion in the same crystal is one of the fundamental goals in the field. This requires a rational design of the synthesis at macroscopic scale and precise control of essential elusive SCO parameters, such as critical temperature (*T*_1/2_), abruptness, hysteresis width, and completeness. Relevant achievements of this strategy include the combination of SCO and nonlinear optical properties,^4^ electronic conduction,^5^ electroluminescence,^6^ fluorescence,^7^ liquid-crystalline properties,^8^ porosity,^2d,2g^ molecular recognition,^9^ photoswitchable magnets,^10^ chirality,^11^ room-temperature photoisomers and reactions,^12^ etc. The ultimate goal is the construction of sensing materials capable of acting as switchers in response to changes of ambient conditions (temperature, humidity, chemical contaminants, etc.). Furthermore, the potential implementation of SCO materials into electronic and spintronic devices is a new concept of paramount importance that has fuelled sophisticated studies aiming at controlling the electron transport (charge and spin) processing SCO materials as ultrathin films on surfaces.^13^

Two-dimensional (2D) Hofmann-type Fe^II^ coordination polymers with general formula {Fe^II^(L_ax_)_2_[M^II^(CN)_4_]} represent an important source of SCO compounds, where M^II^ = Pt^II^, Pd^II^, or Ni^II^ and L_ax_ is a terminal monotopic axial ligand based on pyridine/pyridine-like^2d,14^ and triazole rings.^15^ The Fe^II^ ions are equatorially connected through square-planar [M^II^(CN)_4_]^2–^ anionic metalloligands affording robust infinite [Fe^II^[M^II^(CN)_4_]_∞_ layers that are the origin of the cooperativity typically exhibited by these compounds. The layers stack on top each other interdigitating the axial ligands L_ax_ whose nature (length, donor–acceptor substituents, etc.) plays an important role in the modulation of the cooperativity through changes in the interlayer spacing and flexibility of the layers (corrugation), factors that may influence the inclusion of guest molecules.

It has recently been shown that 2D Hofmann-type coordination polymers can be processed as ultrathin films under mild conditions (RT) by applying the layer-by-layer liquid phase epitaxy (LPE) methodology,^16−18^ at variance of the homologous 3D derivatives, which require very low temperatures.^19−25^ Processing of these materials as ultrathin films is a requirement to keep small electrode separation in vertical transport devices to ensure a functional current flow but, obviously, it can seriously compromise the SCO properties. For example, synchrotron XAS studies showed that, for film thicknesses above ca. 12 nm, the 2D coordination polymer {Fe^II^(pyridine)_2_[Pt^II^(CN)_4_]} presents a cooperative SCO behavior similar to that observed for the microcrystalline sample.^17^ However, below this threshold value, the cooperativity and completeness of the spin transition are exponentially attenuated since the films lose cohesion conferring to its structure a high degree of mosaicity constituted of practically unconnected nanoislands. The nature of the axial ligand and its dramatic influence on the coalescence of the thin film deposited on Au substrates has also been investigated for two new 2D Hofmann compounds {Fe^II^(pyrimidine)_2_[Pt^II^(CN)_4_]} and {Fe^II^(isoquinoline)_2_[Pt^II^(CN)_4_]}, together with their transport properties.^18^

In the search for new Fe^II^ Hofmann-type 2D coordination polymers, here, we report on the preparation, structural characterization, and spin crossover properties of four complexes generically formulated {Fe^II^(pyS_2_R)_2_[M^II^(CN)_4_]}_*n*_ (**MpyS**_**2**_**R**, where M^II^ = Pd, Pt and R = Me, Et), where the axial organic ligand pyS_2_R is 4-methyl/ethyldisulfanylpyridine (R = Me, Et). In contrast to the mentioned above multilayer studies based of the LPE technique, the axially coordinated pyridine ligand functionalized in 4-position with a reactive alkyldisulfanyl group opens the possibility to process the resulting 2D coordination polymers as robust single monolayer arrays of elastically coupled SCO centers deposited on suitable surfaces to be probed as SCO junctions. This approach was inspired by a relevant pioneer work by Mallouk et al. about the growth of thin films of the porous 3D Hofmann clathrate {Ni(4,4′-bipyridine)[Pt(CN)_4_]} anchored through a monolayer of 4-pyridyl ethyldisulfide on gold substrates.^20^ A similar strategy has recently led to the production of molecular monolayers prepared by simple immersion of the substrate in highly diluted solutions of mononuclear Fe^II^ SCO complexes on gold substrates and successfully tested as spintronic devices.^26^

## Results

### Synthesis

All the samples **MpyS**_**2**_**R** (where M = Pt, Pd and R = Me, Et) were prepared as single crystals from slow diffusion techniques in water–methanol solutions (see the Experimental Section). According to chemical and thermogravimetric analyses (see Figure S1 in the Supporting Information), the single crystals resulted to be unsolvated and decompose above 420 K.

### Spin Crossover Properties

**Figure 1:**
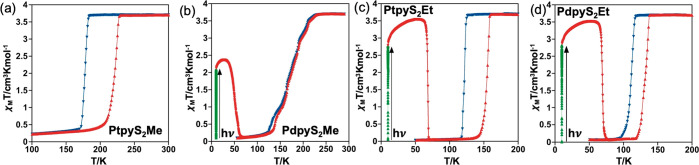
Magnetic and photomagnetic properties of **MpyS**_**2**_**Me** (M = Pt (a), Pd (b)) and **MpyS**_**2**_**Et** (M = Pt (c), Pd (d)). Cooling, heating and photoswitching processes are represented in blue, red, and green, respectively.

Figure 1 shows the magnetic and photomagnetic properties of the title compounds in the form of the product χ_M_*T* vs *T*, where χ_M_ is the molar magnetic susceptibility and *T* is the temperature. At 300 K, the χ_M_*T* value is ca. 3.70 cm^3^ K/mol for the four derivatives consistently with a fully populated HS state with a strong orbital contribution. Upon cooling at 1 K/min, χ_M_*T* remains constant down to 183 K for **PtpyS**_**2**_**Me** and decreases abruptly to 0.4 cm^3^ K/mol in the interval 182–170 K, then decreases gradually to attain a value of 0.2 cm^3^ K/mol at 100 K, where the LS state is practically fully populated. The profile of the χ_M_*T* vs *T* curve in the heating mode is similar to that of the cooling mode but shifted to high temperatures, defining a hysteresis loop Δ*T* = 44 K wide with the equilibrium temperatures, *T*_1/2_, at which the populations of the HS and LS centers are equal at 0.5, equal to 180 K and 224 K for the cooling and heating branches, respectively. This strong cooperative SCO behavior contrasts drastically with that shown by the isostructural (vide infra) homologous **PdpyS**_**2**_**Me** derivative. χ_M_*T* = 3.70 cm^3^ K/mol remains constant down to 232 K; however, below this temperature, it decreases gradually in a succession of slightly marked steps, reaching a value of 0.15 cm^3^ K/mol at 100 K. Except for the lower step, which shows a small hysteresis between 130 K and 138 K, the cooling–heating profiles are practically superposed. The corresponding characteristic *T*_1/2_ temperature is 170 K. The SCO profile for the **MpyS**_**2**_**Et** derivatives is similar to that of **PtpyS**_**2**_**Me**, featuring strong cooperative hysteretic behaviors with *T*_1/2_ temperatures 121.5 and 154.0 K (Δ*T* = 32.5 K) for M = Pt and 111.0 and 132.0 K (Δ*T* = 21.0 K) for M = Pd, in the cooling and heating modes, respectively.

Photogeneration of the fully populated metastable HS* state, the so-called “light-induced excited spin state trapping (LIESST) experiment”,^27^ was performed by irradiating microcrystalline samples of the title compounds with green light (λ = 532 nm) at 10 K. Under these conditions, all the samples but **PtpyS**_**2**_**Me** display the LIESST effect and saturate at χ_M_*T* values of 2.08 cm^3^ K/mol for **PdpyS**_**2**_**Me** and 2.80 cm^3^ K/mol for **MpyS**_**2**_**Et** (M = Pt, Pd). Subsequently, the light was switched off and the temperature increased at a rate of 0.3 K/min inducing a gradual increase of χ_M_*T* to a value of 2.36 cm^3^ K/mol at 26 K for **PdpyS**_**2**_**Me** and 3.54 cm^3^ K/mol at ca. 48 K for **MpyS**_**2**_**Et** (M = Pt, Pd), which corresponds to ca. 64% and 96% of the maximum value observed at 300 K, respectively. This increase in χ_M_*T* reflects the thermal population of different microstates originated from the zero-field splitting of the HS* spin state. At higher temperatures, χ_M_*T* decreases rapidly until joining the thermal SCO curve at ca. 65 K (**PdpyS**_**2**_**Me**), 69 K (**PtpyS**_**2**_**Et**), and 76 K(**PdpyS**_**2**_**Et**), indicating that the metastable HS* state has relaxed back to the stable LS state. The corresponding *T*_LIESST_ temperatures, evaluated as ∂(χ_M_*T*)/∂*T*,^28^ are 50.0 K (**PdpyS**_**2**_**Me**) and 68–70 K (**MpyS**_**2**_**Et**, M = Pt, Pd). These temperatures are consistent with the inverse-energy-gap law, i.e., the metastability of the photogenerated HS* species decreases as the stability of the LS increases, namely as *T*_1/2_ increases.^29^

**Figure 2:**
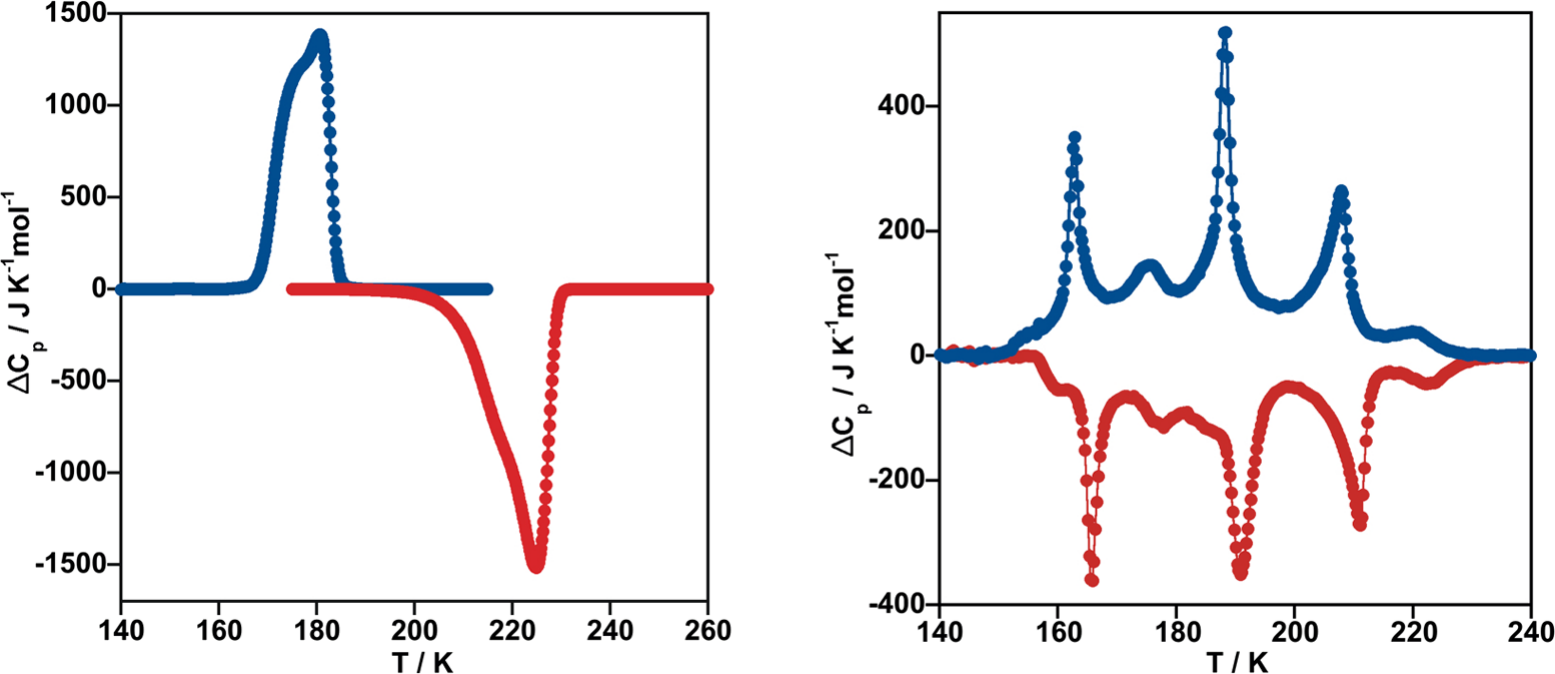
Thermal dependence of Δ*C*_p_ vs T for **PtpyS**_**2**_**Me** (left) and **PdpyS**_**2**_**Me** (right). Note that for the latter the step below 150 K could not be recorded (see text). Cooling and heating modes are represented in blue and red, respectively.

The SCO behavior was also investigated through the thermal dependence of the heat capacity at constant pressure, Δ*C*_p_, for **MpyS**_**2**_**Me** (M = Pt, Pd) (Figure 2). The low SCO temperatures observed for both ethyl derivatives prevented us to evaluate their thermodynamic parameters. The average enthalpy Δ*H* and entropy variations Δ*S* (= Δ*H*/*T*_1/2_) are, respectively, 16.12 kJ/mol and 79.84 J/K mol for **PtpyS**_**2**_**Me** and 7.68 kJ/mol and 45.18 J/K mol for **PdpyS**_**2**_**Me**. The Δ*H* and Δ*S* values found for **PtpyS**_**2**_**Me** are comparable to those reported for similar Hofmann-type coordination polymers with comparable cooperative SCO.^1b,2d^ However, for **PdpyS**_**2**_**Me** these values are considerably smaller due to the fact that ca. 27% of the SCO occurs out of the temperature window of our calorimeter, an extrapolation to 100% gives Δ*H* = 10.5 kJ/mol and Δ*S* = 62 J/Kmol (see also Figure S2 in the Supporting Information). These extrapolated values are still smaller than those observed for **PtpyS**_**2**_**Me** but consistent with the much less cooperative gradual SCO and lower *T*_1/2_ temperature of the homologous Pd derivative. The *T*_1/2_ values obtained from the calorimetric measures are virtually the same than those obtained from magnetism (see Figure S2). As it can be seen from Figure 2, the surprisingly distinct nature of both SCO behaviors, hysteretic versus multistepped, are clearly reflected in the Δ*C*_p_ vs *T* plots.

### Single-Crystal Structure Analysis

#### Structure of **MpyS_2_Me**

**Figure 3:**
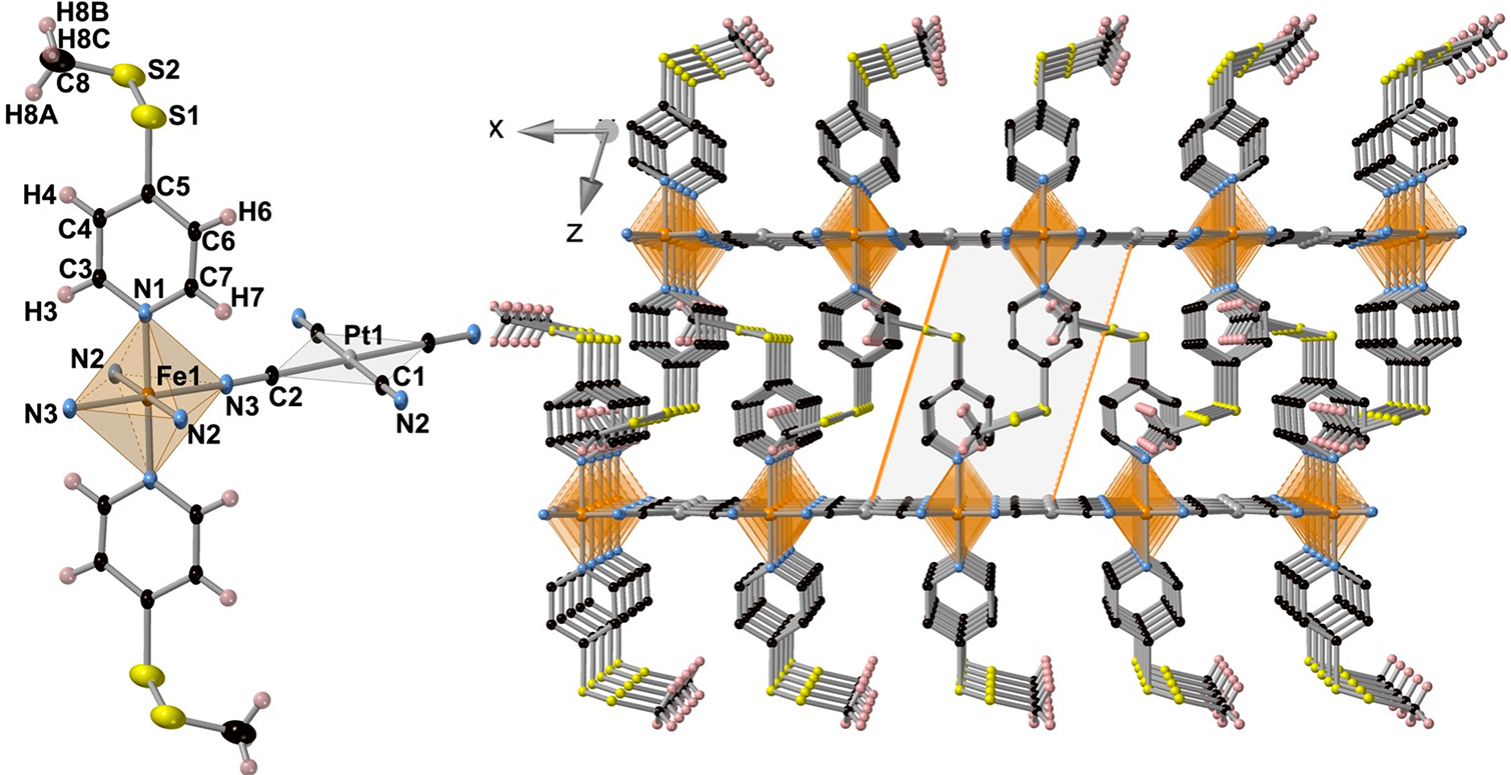
(Left) Molecular fragment of **PtpyS**_**2**_**Me** showing the atom numbering of the asymmetric unit. (Right) Packing of two consecutive layers (only one of the two possible orientations of the −S–S–Me moiety is shown.

The crystal structure of **MpyS**_**2**_**Me** (M = Pt and Pd) was investigated at 120 and 250 K; it turned out to be isostructural and crystallized in the triclinic *P*1̅ space group. A selection of relevant crystallographic data for **MpyS**_**2**_**Me** (M = Pt, Pd) is given in Table S1 in the Supporting Information. At 120 K, the structure is characterized by a crystallographically unique Fe^II^ site lying in an inversion center defining a slightly elongated [Fe^II^N_6_] octahedron. A representative fragment of the structure including the atom numbering is shown in Figure 3 (left). Table 1 contains a selection of significant bond lengths and angles, together with the corresponding average angular distortion parameter Σ^Fe^, which is defined as the sum of deviations from the ideal octahedron/tetrahedron of the 12 “cis” bond angles, ∑_12_^*i*=1^ |θ*_i_* – 90°|. The equatorial positions are occupied by the N2 and N3 atoms of the CN groups belonging to the [Pt^II^(CN)_4_]^2–^ bridging ligands, while the axial positions are occupied by the N1 atom of the pyridine group of the pyS_2_Me ligand. The average ⟨Fe–N⟩ bond length, 1.960(14) Å (M = Pt) and 1.957(3) Å (M = Pd), are typical of the Fe^II^ site in the LS state and consistent with the magnetic data and the characteristic deep red color of the crystals at same temperature. The Σ^Fe^ parameter, almost 0 for the Pd derivative and relatively much larger for the Pt derivative, denote that the angular distortion in both compounds is very small and practically independent of the spin state.

**Table 1:** Selected Bond Lengths and Angles for **MpyS**_**2**_**Me** (M = Pd, Pt)

	PdpyS_2_Me, 120 K	PdpyS_2_Me, 250 K	PtpyS_2_Me, 120 K	PtpyS_2_Me, 250 K
**Selected Bond Lengths [Å]**				
Fe–N(1)	1.987(3)	2.219(5)	1.992(14)	2.23(3)
Fe–N(2)	1.942(3)	2.132(4)	1.943(14)	2.13(2)
Fe–N(3)	1.943(3)	2.137(4)	1.945(14)	2.11(2)
Pd–C(1)	1.991(3)	1.991(4)		
Pd–C(2)	1.992(3)	1.988(5)		
Pt–C(1)			1.975(17)	1.97(2)
Pt–C(2)			1.978(17)	1.93(2)
C(1)–N(2)	1.149(4)	1.131(7)	1.15(3)	1.16(3)
C(2)–N(3)	1.153(5)	1.130(7)	1.15(3)	1.21(3)

**Selected Bond Angles (°)**				
N(1)–Fe–N(2)	90.06(12)	90.1(2)	90.7(6)	91.6(11)
N(1)–Fe–N(3)	90.13(12)	90.0(2)	90.5(6)	91.0(11)
N(2)–Fe–N(3)	90.01(10)	90.04(14)	91.0(6)	90.2(6)
Σ^Fe^	0.8	0.56	8.8	11.2
C(1)–N(2)–Fe	179.8(2)	179.7(5)	178(2)	169(2)
C(2)–N(3)–Fe	179.8(3)	179.9(5)	177(2)	178(2)

Each Fe^II^ site is bridged to four equivalent Fe^II^ sites through four equivalent square-planar [Pt^II^(CN)_4_]^2–^ bridges defining 2D layers in which the equatorial planes of the [Fe^II^N_6_] and [Pt^II^C_4_] centers are strictly coplanar (Figure 3, right). Two consecutive layers interdigitate in such a way that the pyS_2_Me axial ligands of one layer point toward the center of the square windows of the adjacent layers, with the distance between the {Fe^II^_2_[M^II^(CN)_4_]_2_}_*n*_ layers being equal to 10.36 Å (M = Pt) and 10.75 Å (M = Pd). The S–S–CH_3_ tails display positional disorder in two equivalent positions for the Pt derivative while the disorder is considerably more severe for the Pd derivative also involving the pyridine groups (see Figure S3 in the Supporting Information). At 250 K, the structures are essentially the same, being the most significant differences, with respect to those at 120 K, the increase of the ⟨Fe–N⟩ bond length by 0.2 Å and the change of color of the crystals to yellow. Both facts are perfectly consistent with the full population of the Fe^II^ HS state in agreement with the magnetic data. In addition, the change to the HS state in the Pt derivative is accompanied by a small degree of corrugation. The angle defined between the equatorial Fe^II^N_4_ and the [Pt^II^(CN)_4_]^2–^ square planes is 7.64°. Consistently, the Fe–N2–C1 angle decreases 9° from 178(2)° in the LS state until 169(2)° in the HS state. In addition, the separation of two consecutive [Fe_2_M_2_]_n_ layers increases by 0.38 Å until 10.74 Å. In contrast, the layers remain perfectly flat for the Pd derivative while the separation between consecutive layers increases by 0.2 Å until 10.95 Å (see Figure S2).

#### Structure of **MpyS_2_Et**

**Figure 4:**
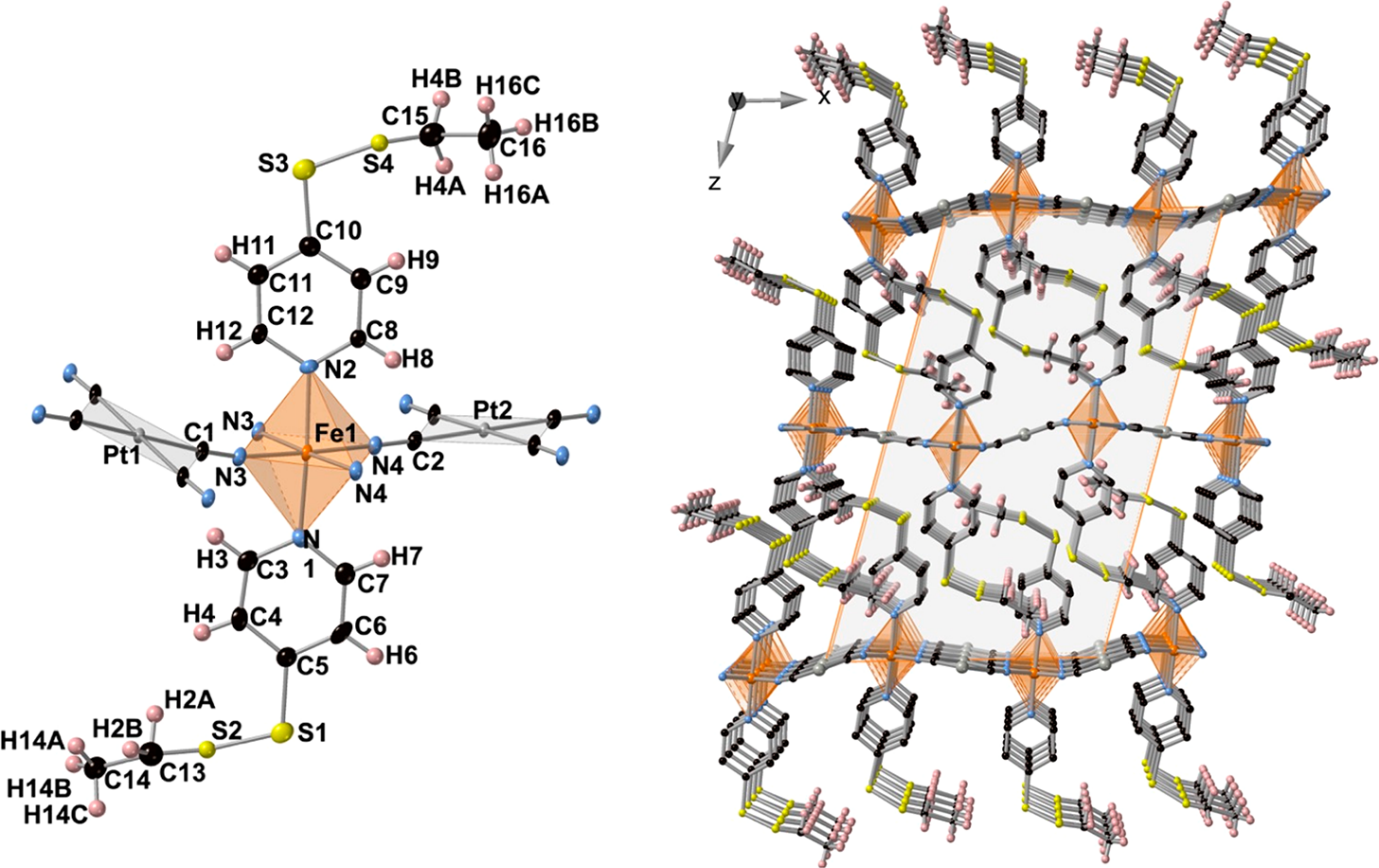
(Left) Molecular fragment of **PtpyS**_**2**_**Et** showing the atom numbering of the asymmetric unit. (Right) Packing of three consecutive layers (only one of the two possible orientations of the −S–S–Et moiety is shown.

The crystal structures of **MpyS**_**2**_**Et**, M = Pt and Pd, were investigated at 100 and 250 K turning out to be isostructural. At 100 K, the red crystals of both derivatives display a monoclinic *I*2/*m* unit cell that changes to monoclinic *C*2/*m* at 250 K, where the crystals are yellow (see Table S2). Table 2 contains a selection of significant bond lengths and angles including the angular distortion parameter Σ^Fe^. The asymmetric unit contains one slightly distorted [Fe^II^N_6_] octahedral site defined by two distinct pyS_2_Et axial ligands coordinated, respectively, via N1 and N2 and two distinct [M(CN)_4_]^2–^ groups coordinated, respectively, via N3 and N4 (Figure 4). The two pyridine rings of pyS_2_Et and the Fe^II^ center lie in a reflection plane which bisects the equatorial N3–Fe–N3′ and N4–Fe–N4′ angles. At 100 K, the ⟨Fe–N⟩ is 1.961(5) and 1.968(4) Å for the Pt and Pd derivatives, respectively, are consistent with the Fe^II^ centers in a fully populated LS state, whereas, at 250 K, these average bond lengths increase by 0.20–0.21 Å attaining typical values for the Fe^II^ in the HS state [2.166(9) and 2.179(9) Å, respectively]. The Σ^Fe^ parameter is small (∼20°) and remains almost constant upon SCO. There are two crystallographically distinct [M^II^(CN)_4_]^2–^ groups and two pairs of them connect each Fe^II^ center to four equivalent atoms defining an irregularly corrugated layer. Indeed, at 100 K, the angle defined between the [M1^II^(CN)_4_]^2–^/[M2^II^(CN)_4_]^2–^ squares and the equatorial plane of the Fe^II^ center is, respectively, 4.04°/4.24° and 20.47°/21.40° and increase by ca. 36% up to 6.22°–6.56° and 32.26°–35.82° for M = Pt/Pd at 250 K. The change of this angle occurs through the Fe–N3–C1, which decrease 10.9° (Pd) and 9° (Pt) when moving from the LS to the HS state. The separation between two consecutive the layers, measured from the average plane defined by M1 and M2, is very similar for the two derivatives and practically does not change with temperature (11.15–11.52 Å).

**Table 2:** Selected Bond Lengths and Angles for **MpyS**_**2**_**Et** (M = Pd, Pt)

	PdpyS_2_Et, 100 K	PdpyS_2_Et, 250 K	PtpyS_2_Et, 100 K	PtpyS_2_Et, 250 K
**Selected Bond Lengths [Å]**				
Fe–N(1)	2.001(5)	2.218(8)	1.997(7)	2.205(7)
Fe–N(2	2.017(5)	2.237(9)	2.004(6)	2.202(9)
Fe–N(3)	1.946(4)	2.170(5)	1.940(5)	2.162(5)
Fe–N(4)	1.948(4)	2.140(5)	1.944(5)	2.133(5)
Pd(1)–C(1)	1.992(4)	1.987(7)		
Pd(2)–C(2)	2.003(5)	2.005(6)		
Pt(1)–C(1)			1.981(5)	1.987(5)
Pt(2)–C(2)			1.989(5)	1.993(5)
C(1)–N(3)	1.146(5)	1.145(8)	1.160(7)	1.142(6)
C(2)–N(4)	1.146(5)	1.126(8)	1.159(7)	1.141(7)

**Selected Bond Angles [°]**				
N(1)–Fe–N(3)	91.29(14)	90.7(2)	91.4(2)	90.5(2)
N(1)–Fe–N(4)	90.72(14)	91.3(2)	90.6(2)	91.2(2)
N(2)–Fe–N(3)	86.83(14)	86.7(2)	86.6(2)	86.6(2)
N(2)–Fe–N(4)	91.18(14)	91.2(2)	91.5(2)	91.7(2)
N(3)–Fe–N(4)	88.43(14)	88.3(2)	88.3(2)	88.5(2)
N(3)–Fe–N(3)	91.9(2)	90.4(3)	92.0(3)	90.2(3)
N(4)–Fe–N(4)	91.2(2)	93.0(3)	91.3(3)	92.7(3)
Σ	19.96	19.8	20.5	19.5
C(1)–N(3)–Fe	169.7(4)	158.8(6)	169.3(4)	160.3(5)
C(2)–N(4)–Fe	178.0(3)	177.7(6)	178.1(4)	177.1(5)

## Discussion

Since the first SCO Hofmann type 2D coordination polymer {Fe^II^(pyridine)_2_[M^II^(CN)_4_]}, M^II^ = Ni,^30^ and its isostructural Pd^II^ and Pt^II^ counterparts^31^ were reported, this family of compounds has been steadily growing until recently (see refs (2d, 2g, 14, 15, 18)). Despite their high insolubility, their synthesis based on formal replacement of the axial pyridines with homologous N-donor ligands can be addressed in a straightforward manner to obtain samples constituted exclusively of single crystals by employing liquid–liquid slow diffusion techniques, which is the safest way to get pure samples with reliable SCO properties for this type of compounds. The strong cooperative SCO behavior featuring well-shaped symmetric hysteresis loops expressed by many of these compounds is likely the most appealing aspect, which explains the interest and growth of this family of compounds. This cooperativity seems to be originated mainly from the robust nature of the {Fe^II^[M^II^(CN)_4_]}_*n*_ layers where all SCO centers are strongly coupled. Indeed, most of the [Fe^II^(L)_2_[M^II^(CN)_4_] compounds show hysteresis widths ranging in the interval of 10–30 K, but it has also been reported hysteresis close to 40 K^32a,18b^ or even larger (50–65 K).^15g^ It is reasonable to consider that the nature of the axial ligands and included guest molecules play an important role in the modulation of the observed cooperativity, however, this is a fact that generally has not obvious rationalization. In addition, it is also worth noting that the hysteresis width seems not to be correlated with the length of the axial ligand, namely, with the separation between the {Fe^II^[M^II^(CN)_4_]}_*n*_ layers. For example, interdigitation of the relatively long ligands L = 4-styrylpyridine (*n* = 0.5) and 4-(2-phenylethyl)pyridine (*n* = 0) in [Fe^II^(L)_2_[Pt^II^(CN)_4_]·*n*MeOH separates the {Fe^II^[Pt^II^(CN)_4_]}_*n*_ layers by ca. 13.85 Å and although both compounds display sharp SCO transitions they lack of hysteretic behavior.^32b^ In contrast, the closely related axial ligands L = 3-phenylazo-pyridine and 4-phenylazopyridine in [Fe^II^(L)_2_[Pd^II^(CN)_4_] with similar interdigitation induce abrupt hysteretic spin transitions with Δ*T*_1/2_ = 12 and 17 K, respectively.^32c^ An additional difficulty when dealing with this type of compounds is that the spin crossover nature (*T*_1/2_, Δ*T*_1/2_, completeness, abruptness, etc.) may be strongly affected by the degree of crystallinity. A relevant example has been recently observed for {Fe^II^(pyridine)_2_[Pt^II^(CN)_4_]} (separation between layers {Fe^II^[Pt^II^(CN)_4_]}_*n*_ ca. 7.6 Å). In its precipitated microcrystalline form, it displays a SCO centered at 212 K with a hysteresis 8 K wide, which is characterized by a remarkable residual fraction (15%–19%) of inactive HS centers. In contrast, the same compound exclusively constituted of single crystals shows a complete well-shaped SCO centered at 234 K and a hysteresis 42 K wide (see Figure S4 in the Supporting Information).^17^ Rapid precipitation of these highly insoluble compounds usually produces microcrystalline samples consisting of submicrometric/nanometric crystallites, dramatically influencing the SCO via the increase of crystal defects, and hence consisting of the residual HS molar fraction in the LS phase, which, in turn, is reflected on a decrease of the *T*_1/2_, of cooperativity (Δ*T*_1/2_) and completeness of the SCO.

In the present study, the SCO behavior has been investigated for samples exclusively constituted of single crystals. Except for **PdpyS**_**2**_**Me**, the SCO behavior of the title compounds **MpyS**_**2**_**R** (R = Me, Et; M = Pd, Pt) retain the general features described for other Hofmann-type 2D coordination polymers. Compound **PtpyS**_**2**_**Me** undergoes a particularly strong cooperative transition with a hysteresis Δ*T* = 44 K wide, which, despite an interlayer distance increase of ∼2–3 Å, because of the presence of the flexible −S–S–CH_3_ moieties, it is virtually the same than the SCO observed for single crystals of {Fe^II^(pyridine)_2_[Pt^II^(CN)_4_]}. The only noticeable difference is observed for the average *T*_1/2_^av^ value, which is 32 K less than that observed for the pyridine derivative. This result also supports the idea mentioned above that separation between the layers does not substantially affect the cooperativity.

Replacement of the methyl group by the ethyl group in **MpyS**_**2**_**R** does not change significantly the separation between the layers but involves a considerable decrease in *T*_1/2_^av^ from 202 K to 138 K (64 K) for the Pt derivative. This fact could tentatively be correlated with a higher corrugation of the layers in the ethyl derivatives. This fact is clearly reflected in the decrease from 180° of one of the two Fe–N–C–Pt moieties. For **PtpyS**_**2**_**Me**, the angle Fe–N2–C1(Pt) is 168.5°, while the equivalent angle for **PtpyS**_**2**_**Et**, Fe–N3–C1(Pt), is 158.8°, both in the HS state, and they change to 178.0° and 169.6° in the LS state, respectively. Obviously, the larger misalignment of the N–C–Pt moiety, with respect to the 3d orbitals of Fe^II^ in the ethyl derivative, must necessarily decrease the σ and π overlaps, thereby decreasing the ligand field felt by the Fe^II^ centers. Another important difference pointing to the same direction is that the angular distortion Σ^Fe^ (see Tables 1 and 2) is significantly larger for **PtpyS**_**2**_**Et** than for its methyl counterpart.

Surprisingly, even though both **MpyS**_**2**_**Me** (M = Pd, Pt) compounds are isostructural, their SCO properties are drastically different to each other. The Pd derivative shows a relatively gradual multistep behavior (ca. 6 steps) separated by very narrow plateaus, while the Pt derivative displays a sharp cooperative spin transition with large hysteresis. The most significant structural difference between them is the occurrence of positional disorder of the pyridine and S–S–CH_3_ groups over four orientations in the Pd derivative, which remains in the HS and LS states. This behavior is reminiscent of that found, among others, for the 2D coordination polymer {Fe^II^[Hg^II^(SCN)_3_]_2_(4,4′-bipy)_2_}_n_ where a sequence of different phases characterized by distinct HS/LS fractions and symmetry breaking results from competition between SCO and structural 4,4′-bipy ligand ordering. For this system, it was possible to identify a correlation between the internal dihedral angle adopted by the 4,4′-bipy ligand and each particular step (spin state phase) as being responsible for the observed multistability.^33^ From a phenomenological point of view, thermally induced multistep SCO behavior is associated with elastic frustration,^3b,3c^ namely, the occurrence of subtle balances between opposed intermolecular interactions that drive the HS ↔ LS transformation in fractional steps consistent with different concentrations of HS and LS centers (with or without ordering). For **PdpyS**_**2**_**Me**, the more conspicuous positional disorder may be the source of subtle balances between interlayer interactions and/or distortions of the [FeN_6_] centers. However, to precisely identify the structural constraints favoring the steps, is for most of the known multistep SCO examples a major difficulty in particular when the steps are poorly defined.

## Conclusions

Here, we have described the synthesis, structure, magnetic, photomagnetic, and calorimetric properties of four new Hofmann-type 2D SCO coordination polymers. Three of them show strong cooperative SCO properties, featuring wide thermal hysteresis, in particular compound **PtpyS**_**2**_**Me**, while its isostructural Pd counterpart surprisingly displays a multistepped transition without hysteresis, most likely due to the occurrence of additional disorder in the structure. The **MpyS**_**2**_**Et** derivatives, which have the lowest *T*_1/2_ of the series, show complete LIESST effect. In contrast, the LIESST effect is incomplete for **PdpyS**_**2**_**Me** and vanishes completely for **PtpyS**_**2**_**Me** because of their higher *T*_1/2_ values.

The results here reported correspond to the first step in a more challenging work whose ultimate objective is to graft these Hofmann-type 2D SCO coordination polymers as monolayers on metallic surfaces (e.g., Au) to be probed as junctions for spintronic devices in which the switchable SCO centers can be used to modulate the junction conductance (see Scheme I). The choice of 4-alkyldisulfanylpyridines as axial ligands was based on the well-known fact that S atoms ensure appropriate interaction between the molecular wires and the electrodes. Preliminary work on this second objective confirms its feasibility and definitive conclusions will be reported in due time.

**Scheme I:**
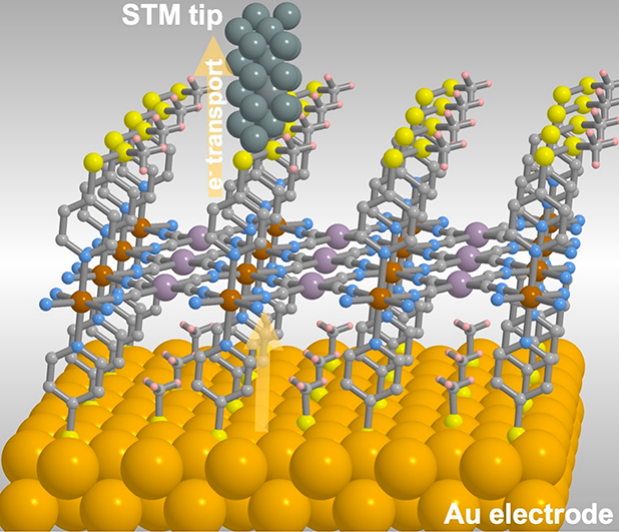
Model of Device Constituted of a Monolayer of **MpyS**_**2**_**Me** Deposited on an Au Substrate

## Experimental Section

### Materials and Reagents

Iron(II) tetrafluoroborate hexahydrate, potassium tetracyanoplatinate(II) trihydrate, potassium tetracyanopalladate(II) hydrate, 4-mercaptopyridine, and methyl methanethiosulfonate were obtained from commercial sources and used as received without further purification. Ethyl methanethiosulfonate was synthesized following a literature procedure.^34^

#### Synthesis of Methyl/Ethyl(4-pyridyl)disulfide

The synthesis of methyl(4-pyridyl)disulfide was performed using a method previously described.^35^ Stoichiometric amounts of NaOH (5 mmol), 4-mercaptopyridine (5 mmol), and methylmethanethiosulfonate (5 mmol) were dissolved in water (10 mL). A white turbidness appears immediately which slowly transforms to a yellow oil. The reaction mixture was stirred at room temperature for 30 min and then extracted with CH_2_Cl_2_. The organic phase was dried with MgSO_4_ and subsequently evaporated to obtain a yellow oil, which was purified by SiO_2_ column chromatography using a toluene-ethyl acetate mixture (80:20) as eluent. 300 MHz, CDCl_3_, δ/ppm: 8.49 (2H), 7.44 (2H), 2.46 (3H). The same route was followed for the synthesis of ethyl(4-pyridyl)disulfide using the noncommercial precursor ethyl methanethiosulfonate. 300 MHz ^1^H-RMN, CDCl_3_, δ/ppm: 8.47 (2H), 7.45 (2H), 2.77 (2H), 1.32 (3H).

#### Synthesis of Complexes **MpyS2R** (M = Pd, Pt; R = Me, Et)

All the samples were constituted of single crystals exclusively obtained through slow liquid-to-liquid diffusion methods using a 10-mL-total-volume H-shaped tube. One arm of the tube was filled with 1 mL of H_2_O:MeOH (1:1) solution containing a mixture of 33.7 mg of Fe(BF_4_)_2_**·**6H_2_O (0.1 mmol) and 40/44 mg (0.25 mmol) of methyl/ethyl(4-pyridyl)disulfide, whereas the other one was filled with an aqueous solution (1 mL) of 44/35 (0.1 mmol) of K_2_[M(CN)_4_] (M = Pt^II^/Pd^II^). The rest of the tube was carefully filled with a methanol:water (1:1) solution, closed with parafilm and left to stand at room temperature. Light yellow cubic single crystals of **MpyS_2_R** were obtained after 2 weeks. Elemental analysis: Calculated for **PtpyS**_**2**_**Me** [C_16_H_14_N_6_S_4_FePt (669.5) (%)]: C 28.70; H 2.11; N 12.55. Found (%): C 29.11; H 2.08; N 12.78. Calculated for **PdpyS**_**2**_**Me** [C_16_H_14_N_6_S_4_FePd (580.8) (%)]: C 33.09; H 2.43; N 14.47. Found (%): C 33.57; H 2.15; N 14.65. Calculated for **PtpyS**_**2**_**Et** [C_18_H_18_N_6_S_4_FePt (697.6) (%)]: C 30.99; H 2.60; N 12.05. Found (%): C 30.52; H 2.52; N 12.35. Calculated for **PdpyS**_**2**_**Et** [C_18_H_18_N_6_S_4_FePd (608.9) (%)]: C 35.51; H 2.98; N 13.80. Found (%): C 35.17; H 2.90; N 14.01.

### Physical Measurements

#### Magnetic Measurements

Variable temperature magnetic susceptibility data were recorded with a Quantum Design MPMS2 SQUID magnetometer equipped with a 7 T magnet, operating at 1 T and at temperatures of 1.8–400 K. Experimental susceptibilities were corrected for diamagnetism of the constituent atoms by the use of Pascal’s constants.

#### Calorimetric Measurements

DSC measurements were performed using a differential scanning calorimeter (Mettler Toledo, Model DSC 821e). Low temperatures were obtained with an aluminum block attached to the sample holder, refrigerated with a flow of liquid nitrogen, and stabilized at a temperature of 110 K. The sample holder was kept in a drybox under a flow of dry nitrogen gas to avoid water condensation. The measurements were performed using ∼15 mg of microcrystalline samples of **MpyS_2_Me** (M = Pt, Pd) sealed in aluminum pans with a mechanical crimp. Temperature and heat flow calibrations were made with standard samples of indium by using its melting transition (429.6 K, 28.45 J g^–1^). An overall accuracy of ±0.2 K in temperature and ±2% in the heat capacity is estimated. The uncertainty increases for the determination of the anomalous enthalpy and entropy due to the subtraction of an unknown baseline.

#### Single Crystal X-ray Diffraction

Single-crystal X-ray data were collected on an Oxford Diffraction Supernova diffractometer using graphite monochromated Mo Kα radiation (λ = 0.71073 Å). A multiscan absorption correction was performed. The structures were solved by direct methods using SHELXS-2014 and refined by full matrix least-squares on *F*^2^ using SHELXL-2014.^36^ Non-hydrogen atoms were refined anisotropically and hydrogen atoms were placed in calculated positions refined using idealized geometries (riding model) and assigned fixed isotropic displacement parameters. CCDC 2072898 (100 K) and 2072899 (250 K) (**PdpyS**_**2**_**Et**); 2072901 (250 K) and 2072902 (129 K) (**PdpyS**_**2**_**Me**); 2072900 (100 K) and 2072905 (250 K) (**PtpyS**_**2**_**Et**); and 2072903 (120 K) and 2072904 (250 K) (**PtpyS**_**2**_**Me**) contain the supplementary crystallographic data for this article. These data can be obtained free of charge from The Cambridge Crystallographic Data Centre via www.ccdc.cam.ac.uk/data_request/cif.

## Supporting Information Available

Relevant crystallographic data (Tables S1 and S2); thermogram for **MpyS**_**2**_**Me** and **MpyS**_**2**_**Et** (M = Pd, Pt) (Figure S1); comparison of the thermal dependence of Δ*C*_p_ and ∂(χ_M_*T*)/∂*T* curves for **PdpyS**_**2**_**Me** (Figure S2); positional disorder of the pyS_2_Me ligands of **PdpyS**_**2**_**Me** at 250 K (Figure S3); comparison of the χ_M_*T* vs *T* plots for {Fe(pyridine)_2_[Pt(CN)_4_]} obtained by direct precipitation and by slow diffusion as single crystals (Figure S4) (PDF)

## Accession Codes

CCDC 2072898–2072905 contain the supplementary crystallographic data for this paper. These data can be obtained free of charge via www.ccdc.cam.ac.uk/data_request/cif, or by emailing data_request@ccdc.cam.ac.uk, or by contacting The Cambridge Crystallographic Data Centre, 12 Union Road, Cambridge CB2 1EZ, UK; fax: +44 1223 336033.

## References

[ref1] aReal J. A.; Gaspar A. B.; Niel V.; Muñoz M. C. Communication between iron(II) building blocks in cooperative spin transition phenomena. Coord. Chem. Rev. 2003, 236, 121–141. 10.1016/S0010-8545(02)00220-5.

[ref2] aWeber B. Spin crossover complexes with N_4_O_2_ coordination sphere-The influence of covalent linkers on cooperative interactions. Coord. Chem. Rev. 2009, 253, 2432–2449. 10.1016/j.ccr.2008.10.002.

[ref3] aBertoni R.; Lorenc M.; Tissot A.; Boillot M.-L.; Collet E. Femtosecond photoswitching dynamics and microsecond thermal conversion driven by laser heating in Fe^III^ spin-crossover solids. Coord. Chem. Rev. 2015, 282-283, 66–76. 10.1016/j.ccr.2014.05.024.

[ref4] Lacroix P. G.; Malfant I.; Real J.-A.; Rodriguez V. From magnetic to nonlinear optical switches in spin-crossover complexes. Eur. J. Inorg. Chem. 2013, 2013, 615–627. 10.1002/ejic.201201151.

[ref5] Lefter C.; Davesne V.; Salmon L.; Molnár G.; Demont P.; Rotaru A.; Bousseksou A. Charge Transport and Electrical Properties of Spin Crossover Materials: Towards Nanoelectronic and Spintronic Devices. Magnetochemistry 2016, 2, 18 10.3390/magnetochemistry2010018.

[ref6] Matsuda M.; Isozaki H.; Tajima H. Reproducible on–off switching of the light emission from the electroluminescent device containing a spin crossover complex. Thin Solid Films 2008, 517, 1465–1467. 10.1016/j.tsf.2008.09.034.

[ref7] aGarcia J.; Robert F.; Naik A. D.; Zhou G.; Tinant B.; Robeyns K.; Michotte S.; Piraux L. Spin transition charted in a fluorophore-tagged thermochromic dinuclear iron(II) complex. J. Am. Chem. Soc. 2011, 133, 15850–15853. 10.1021/ja205974q. 21936512

[ref8] Gaspar A. B.; Seredyuk M. Spin crossover in soft matter. Coord. Chem. Rev. 2014, 268, 41–58. 10.1016/j.ccr.2014.01.018.

[ref9] aNi Z.; Shores M. P. Magnetic observation of anion binding in iron coordination complexes: Toward Spin-Switching Chemosensors. J. Am. Chem. Soc. 2009, 131, 32–33. 10.1021/ja807379a. 19072046

[ref10] aOhkoshi S.-I.; Imoto K.; Tsunobuchi Y.; Takano S.; Tokoro H. Light-induced spin-crossover magnet. Nat. Chem. 2011, 3, 564–569. 10.1038/nchem.1067. 21697879

[ref11] aBartual-Murgui C.; Piñeiro-López L.; Valverde-Muñoz F. J.; Muñoz M. C.; Seredyuk M.; Real J. A. Chiral and racemic spin crossover polymorphs in a family of mononuclear iron(II) compounds. Inorg. Chem. 2017, 56, 13535–13546. 10.1021/acs.inorgchem.7b02272. 29048915

[ref12] aVenkataramani S.; Jana U.; Dommaschk M.; Sönnichsen F. D.; Tuczek F.; Herges R. Magnetic bistability of molecules in homogeneous solution at room temperature. Science 2011, 331, 445–448. 10.1126/science.1201180. 21273483

[ref13] aSenthil Kumar K.; Ruben M. Emerging trends in spin crossover (SCO) based functional materials and devices. Coord. Chem. Rev. 2017, 346, 176–205. 10.1016/j.ccr.2017.03.024.

[ref14] aLiu W.; Wang L.; Su Y.-J.; Chen Y. C.; Tucek J.; Zboril R.; Ni Z.-P.; Tong M.-L. Hysteretic Spin Crossover in Two-Dimensional (2D) Hofmann-Type Coordination Polymers. Inorg. Chem. 2015, 54, 8711–8716. 10.1021/acs.inorgchem.5b01341. 26258593

[ref15] aKlein Y. M.; Sciortino N. F.; Ragon F.; Housecroft C. E.; Kepert C. J.; Neville S. M. Spin crossover intermediate plateau stabilization in a flexible 2-D Hofmann-type coordination polymer. Chem. Commun. 2014, 50, 3838–3840. 10.1039/C4CC01079E. 24589976

[ref16] Sakaida S.; Otsubo K.; Sakata O.; Song C.; Fujiwara A.; Takata M.; Kitagawa H. Crystalline coordination framework endowed with dynamic gate-opening behaviour by being downsized to a thin film. Nat. Chem. 2016, 8, 377–383. 10.1038/nchem.2469. 27001734

[ref17] Rubio-Giménez V.; Bartual-Murgui C.; Galbiati M.; Núñez-López A.; Castells-Gil J.; Quinard B.; Seneor P.; Otero E.; Ohresser P.; Cantarero A.; Coronado E.; Real J. A.; Mattana R.; Tatay S.; Martí-Gastaldo S. Effect of nanostructuration on the spin crossover transition in crystalline ultrathin films. Chem. Sci. 2019, 10, 4038–4047. 10.1039/C8SC04935A. 31015944 PMC6460953

[ref18] aRubio-Giménez V.; Escorcia-Ariza G.; Bartual-Murgui C.; Sternemann C.; Galbiati M.; Castells-Gil J.; Real J. A.; Tatay S.; Martí-Gastaldo C. Ultrathin films of 2D Hofmann-type coordination polymers: influence of pillaring linkers on structural flexibility and vertical charge transport. Chem. Mater. 2019, 31, 7277–7287. 10.1021/acs.chemmater.9b01634.

[ref19] Otsubo K.; Haraguchi T.; Kitagawa H. Nanoscale crystalline architectures of Hofmann-type metal–organic frameworks. Coord. Chem. Rev. 2017, 346, 123–138. 10.1016/j.ccr.2017.03.022.

[ref20] Bell C. M.; Arendt M. F.; Gomez L.; Schmehl R. H.; Mallouk T. E. Growth of lamellar Hofmann clathrate films by sequential ligand exchange reactions: assembling a coordination solid one layer at a time. J. Am. Chem. Soc. 1994, 116, 8374–8375. 10.1021/ja00097a058.

[ref21] Cobo S.; Molnár G.; Real J. A.; Bousseksou A. Multilayer sequential assembly of thin films that display room-temperature spin crossover with hysteresis. Angew. Chem., Int. Ed. 2006, 45, 5786–5789. 10.1002/anie.200601885. 16871608

[ref22] Otsubo K.; Haraguchi T.; Sakata O.; Fujiwara A.; Kitagawa H. Step-by-step fabrication of a highly oriented crystalline three-dimensional pillared-layer-type metal-organic framework thin film confirmed by synchrotron X-Ray diffraction. J. Am. Chem. Soc. 2012, 134, 9605–9608. 10.1021/ja304361v. 22650356

[ref23] Bartual-Murgui C.; Salmon L.; Akou A.; Thibault C.; Molnár G.; Mahfoud T.; Sekkat Z.; Real J. A.; Bousseksou A. High quality nano-patterned thin films of the coordination compound {Fe(Pyrazine)[Pt(CN)4]} deposited layer-by-layer. New J. Chem. 2011, 35, 2089–2094. 10.1039/c1nj20212j.

[ref24] Bartual-Murgui C.; Akou A.; Salmon L.; Molnár G.; Thibault C.; Real J. A.; Bousseksou A. Guest effect on nanopatterned spin-crossover thin films. Small 2011, 7, 3385–3391. 10.1002/smll.201101089. 21997948

[ref25] Agusti G.; Cobo S.; Gaspar A. B.; Molnar G.; Moussa N. O.; Szilagyi P. A.; Palfi V.; Vieu C.; Munoz M. C.; Real J. A.; Bousseksou A. Thermal and light-induced spin crossover phenomena in new 3D Hofmann-like microporous metalorganic frameworks produced as bulk materials and nanopatterned thin films. Chem. Mater. 2008, 20, 6721–6732. 10.1021/cm8019878.

[ref26] aAragones A. C.; Aravena D.; Cerda J. I.; Acis-Castillo Z.; Li H.; Real J. A.; Sanz F.; Hihath J.; Ruiz E.; Diez-Perez I. Large conductance switching in a single-molecule device through room temperature spin-dependent transport. Nano Lett. 2016, 16, 218–226. 10.1021/acs.nanolett.5b03571. 26675052

[ref27] Decurtins S.; Gütlich P.; Köhler P. C.; Spiering H.; Hauser A. Light-induced excited spin state trapping in a transition-metal complex: The hexa-1-propyltetrazole-iron (II) tetrafluoroborate spin-crossover system. Chem. Phys. Lett. 1984, 105, 1–4. 10.1016/0009-2614(84)80403-0.

[ref28] Létard J. F.; Guionneau P.; Rabardel L.; Howard J. A. K.; Goeta A. E.; Chasseau D.; Kahn O. Structural, magnetic, and photomagnetic studies of a mononuclear iron(II) derivative exhibiting an exceptionally abrupt spin transition. light-induced thermal hysteresis phenomenon. Inorg. Chem. 1998, 37, 4432–4441. 10.1021/ic980107b. 11670580

[ref29] aHauser A. Intersystem crossing in Fe(II) coordination compounds. Coord. Chem. Rev. 1991, 111, 275–290. 10.1016/0010-8545(91)84034-3.

[ref30] Kitazawa T.; Gomi Y.; Takahashi M.; Takeda M.; Enomoto M.; Miyazaki A.; Enoki T. Spin-crossover behaviour of the coordination polymer Fe^II^(C_5_H_5_N)_2_Ni^II^(CN)_4_. J. Mater. Chem. 1996, 6, 119–121. 10.1039/jm9960600119.

[ref31] Niel V.; Martinez-Agudo J. M.; Muñoz M. C.; Gaspar A. B.; Real J. A. Cooperative spin crossover behavior in cyanide-bridged Fe(II)-M(II) bimetallic 3D Hofmann-like networks (M = Ni, Pd, and Pt). Inorg. Chem. 2001, 40, 3838–3839. 10.1021/ic010259y. 11466039

[ref32] aSeredyuk M.; Gaspar A. B.; Ksenofontov V.; Verdaguer M.; Villain F.; Gutlich P. Thermal- and light-induced spin crossover in novel 2D Fe(II) metalorganic frameworks {Fe(4PhPy)_2_[M^II^(CN)_x_]_y_}·sH_2_O: Spectroscopic, Structural, and Magnetic Studies. Inorg. Chem. 2009, 48, 6130–6141. 10.1021/ic900491r. 19462941

[ref33] aTrzop E.; Zhang D.; Piñeiro-Lopez L.; Valverde-Muñoz F. J.; Muñoz M. C.; Palatinus L.; Guerin L.; Cailleau H.; Real J. A.; Collet E. First step towards a devilÏs staircase in spin-crossover materials. Angew. Chem., Int. Ed. 2016, 55, 8675–8679. 10.1002/anie.201602441. 27193972

[ref34] Bentley M.; Douglass I.; Lacadie J. A. Silver-assisted displacements on sulfur. New thiolsulfonate ester synthesis. J. Org. Chem. 1972, 37, 333–334. 10.1021/jo00967a040.

[ref35] Kitson T. M.; Loomes K. M. Synthesis of methyl 2- and 4-pyridyl disulfide from 2- and 4-thiopyridone and methyl methanethiosulfonate. Anal. Biochem. 1985, 146, 429–430. 10.1016/0003-2697(85)90563-9. 4025805

[ref36] Sheldrick G. M. Crystal Structure Refinement with SHELXL. Acta Crystallogr., Sect. C: Struct. Chem. 2015, 71, 3–8. 10.1107/S2053229614024218. 25567568 PMC4294323

