# Supplementary material for: Bistable Hofmann-Type FeII Spin-Crossover Two-Dimensional Polymers of 4-Alkyldisulfanylpyridine for Prospective Grafting of Monolayers on Metallic Surfaces
Source: Inorg Chem. 2021 May 28;60(12):9040–9. doi: 10.1021/acs.inorgchem.1c01010 (PMC9129067; doi:10.1021/acs.inorgchem.1c01010)

Supporting Information

Bistable Hofmann-type Fe^{II} spin-crossover two-dimensional polymers of 4-alkyldisulfanylpyridine for prospective grafting of monolayers on metallic surfaces

Rubén Turo-Cortés,[a] Francisco Javier Valverde-Muñoz,[a]* Manuel Meneses-Sánchez,[a] M. Carmen Muñoz,[b] Carlos Bartual-Murgui,[a] José Antonio Real[a]*

[a]Instituto de Ciencia Molecular/Departamento de Química Inorgánica, Universidad de Valencia, Catedrático Beltrán Martínez 2, 46980 Paterna, Valencia, Spain.
[b] Departamento de Física Aplicada, Universitat Politècnica de València, Camino de Vera S/N 46022 Valencia, Spain

Email: francisco.valverde@uv.es.
Email: jose.a.real@uv.es.

Page 1- **Table S1**. Crystallographic data for **MpyS$_2$Me** (M = Pd, Pt).

Page 2- **Table S2**. Crystallographic data for **MpyS$_2$Et** (M = Pd, Pt).

Page 3- **Figure S1**. Thermogravimetric analysis of **MpyS$_2$R** (M = Pd, Pt; R = Me, Et).

Page 4- **Figure S2**. Comparison of the thermal dependence of ΔC_p and $\partial(\chi_M T)/\partial T$ curves for **PdpyS$_2$Me**.

Page 4- **Figure S3**. Perspective view of **PdpyS$_2$Me** at 250K showing the perfectly flat $\{Fe[Pd(CN)_4]_n$ layers and the positional disorder of the pyS$_2$Me ligands over four positions.

Page 5- **Figure S4**. Comparison of the $\chi_M T$ vs T plots for $\{Fe(pyridine)_2[Pt(CN)_4]\}$ obtained by direct precipitation and by slow diffusion as single crystals.

Table S1. Crystal data for **$MpyS_2Me$** (M = Pd, Pt).

	**$PdpyS_2Me$_120K**	**$PdpyS_2Me$_250K**	**$PtpyS_2Me$_120K**	**$PtpyS_2Me$_250K**
Empirical formula	$C_{16}H_{14}N_6S_4PdFe$		$C_{16}H_{14}N_6S_4PtFe$	$C_{16}H_8N_6S_4PtFe$
*M*r	580.82		669.51	663.46
Crystal system	triclinic			
Space group	*P*-1			
a (Å)	7.189(3)	7.4283(4)	6.9945(8)	7.2382(8)
b (Å)	7.192(3)	7.4310(4)	7.343(2)	7.591(2)
c (Å)	11.881(5)	12.1476(8)	11.065(2)	11.372(3)
α (°)	72.460(19)	107.823(6)	78.58(2)	105.36(2)
β (°)	72.419(14)	107.831(5)	73.11(2)	101.31(2)
γ (°)	89.946(13)	89.990(4)	89.902(13)	90.05(2)
V (Å^3)	555.6(4)	604.45(7)	532.1(2)	589.9(2)
Z	1			
T (K)	120	250	120	250
D_c (mg cm^{-3})	1.736	1.596	2.089	1.867
F(000)	288		320	314
μ (Mo-K$_\alpha$) (mm^{-1})	1.852	1.702	7.655	6.903
Crystal size (mm)	0.01x0.08x0.08		0.05x0.5x0.5	
No. of total reflections	2764	2433	2892	2378
No. of reflections [$I>2\sigma(I)$]	2685	2186	2669	1907
R [$I>2\sigma(I)$]	0.0341	0.0462	0.1234	0.1195
wR [$I>2\sigma(I)$]	0.0838	0.1013	0.3023	0.2940
S	1.109	1.051	1.265	1.154

$R = \Sigma\, ||Fo| - |Fc|| / \Sigma\, |Fo|$; $wR = [\, \Sigma\, [w(Fo^2 - Fc^2)^2] / \Sigma\, [w(Fo^2)^2]]^{1/2}$.
$w = 1/ [\sigma^2(Fo^2) + (m\, P)^2 + n\, P]$ where $P = (Fo^2 + 2Fc^2) / 3$;
m = 0.0393 (**1**), 0.0502 (**2**), 0.2000 (**3**), and 0.2000 (**4**);
n = 0.7715 (**1**), 0.0000 (**2**), 0.0000 (**3**), and 0.0000 (**4**)

Table S2. Crystal data for **MpyS$_2$Et** (M = Pd, Pt).

	**PdpyS$_2$Et_100K**	**PdpyS$_2$Et_250K**	**PtpyS$_2$Et_100K**	**PtpyS$_2$Et_250K**
Empirical formula	$C_{18}H_{18}N_6S_4PdFe$	$C_{18}H_{18}N_6S_4PdFe$	$C_{18}H_{18}N_6S_4PtFe$	$C_{18}H_{18}N_6S_4PtFe$
*M*r	608.87	608.87	697.56	697.56
Crystal system	monoclinic	monoclinic	monoclinic	monoclinic
Space group	*I*2/*m*	*C*2/*m*	*I*2/*m*	*C*2/*m*
a (Å)	13.8182(8)	23.9683(14)	13.8437(4)	24.0228(10)
b (Å)	7.3599(4)	7.6791(2)	7.3489(2)	7.6448(2)
c (Å)	23.201(2)	13.8679(7)	23.3286(8)	13.9959(5)
β (°)	106.079(7)	106.053(5)	106.218(3)	106.485(4)
V (Å^3)	2267.2(3)	2452.9(2)	2278.91(12)	2464.7(2)
Z	4		4	
T (K)	100	250	100	250
D_c (mg cm^{-3})	1.784	1.649	2.033	1.880
F(000)	1216	1216	1344	1344
μ (Mo-K$_\alpha$) (mm^{-1})	1.820	1.682	7.153	6.614
Crystal size (mm)	0.1x0.5x0.5		0.1x0.7x0.7	
No. of total reflections	3166	3398	3272	3489
No. of reflections [I>2σ(I)]	2179	2052	2611	2588
R [I>2σ(I)]	0.0520	0.0707	0.0332	0.0369
wR [I>2σ(I)]	0.0862	0.1355	0.0832	0.0887
S	1.035	1.035	1.119	1.064

$R = \Sigma\,||Fo| - |Fc|| / \Sigma\,|Fo|$; $wR = [\,\Sigma\,[w(Fo^2 - Fc^2)^2] / \Sigma\,[w(Fo^2)^2]]^{1/2}$.
$w = 1/ [\sigma^2(Fo^2) + (\mathrm{m}\,P)^2 + \mathrm{n}\,P]$ where $P = (Fo^2 + 2Fc^2) / 3$;
m = 0.0275 (**1**), 0.0430 (**2**), 0.0346 (**3**), and 0.0422 (**4**);
n = 0.0000 (**1**), 23.1512 (**2**), 38.7639 (**3**), and 11.5730 (**4**)

Figure S1. Thermogravimetric analysis of **MpyS$_2$R** (M = Pd, Pt; R = Me, Et).

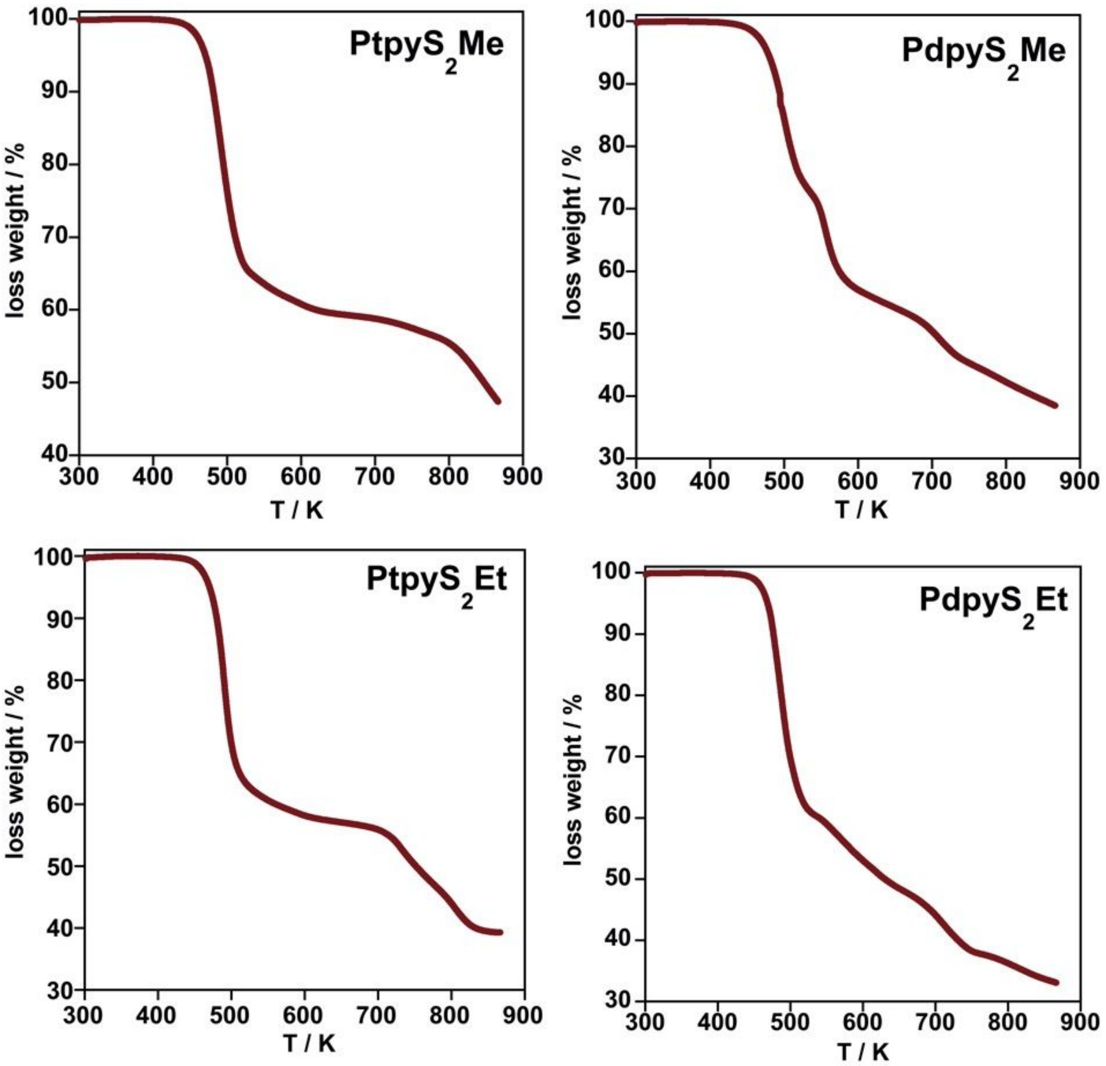

Figure S2. Comparison of the thermal dependence of ΔC_p and $\partial(\chi_M T)/\partial T$ curves for **PdpyS$_2$Me**. Note the excellent match between both curves and the lack in the ΔC_p vs T plot of the peak below 150 K due to experimental constraints.

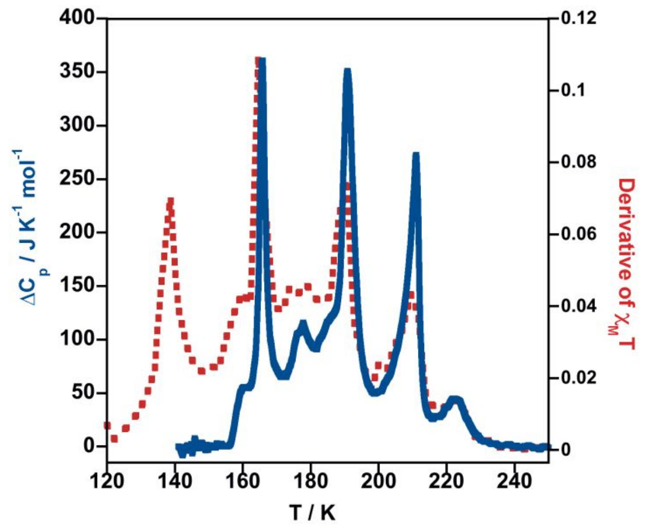

Figure S3. Perspective view of **PdpyS$_2$Me** at 250K showing the perfectly flat {Fe[Pd(CN)$_4$]$_n$ layers and the positional disorder of the pyS$_2$Me ligands over four positions.

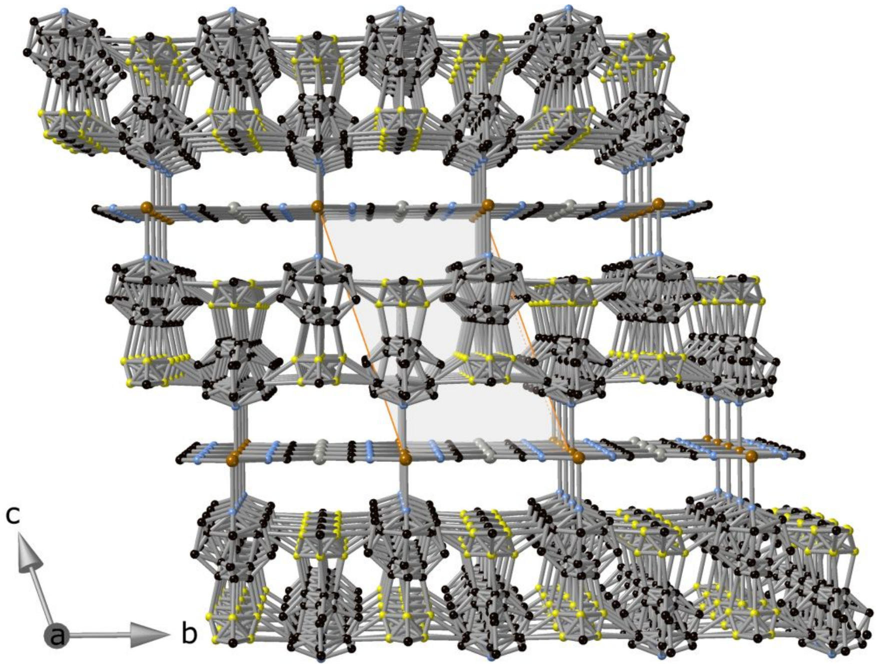

Figure S4. Comparison of the $\chi_M T$ vs T plots for {Fe(pyridine)$_2$[Pt(CN)$_4$]} obtained by direct precipitation as a microcrystalline powder (blue) and by liquid-liquid slow diffusion as single crystals (red).

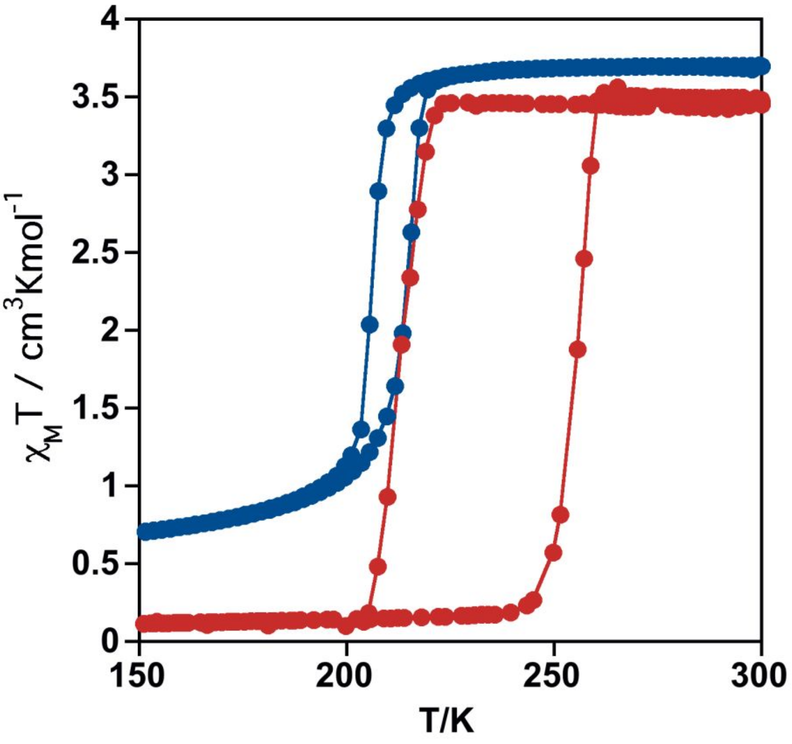

Supplement: Supplementary file 1 — ic1c01010_si_001.pdf [file ic1c01010_si_001.pdf]
